# Bilateral hemotympanum as a result of spontaneous epistaxis

**DOI:** 10.1186/1865-1380-1-3

**Published:** 2011-01-27

**Authors:** Vural Fidan, Kemal Ozcan, Filiz Karaca

**Affiliations:** 1 Ear, Nose and Throat Department, District Education and Research Hospital, 25100 Erzurum, Turkey; 2 Otorhinolaryngology Department, Malatya Government Hospital, Malatya, Turkey; 3 Otorhinolaryngology Department, Erzurum Education and Training Hospital, Erzurum, Turkey

## Abstract

Hemotympanum is a rare condition and usually depends on a secondary reason. Therefore, idiopathic hemotympanum is rarely seen in the literature. In this paper, we report a case of this problem.

## Introduction

Hemotympanum is most often associated with basilar skull fractures or nasal packing. Only six cases associated with spontaneous epistaxis have been described in the literature [1,2]. Because of this rare situation, we present the case of a 51-year-old woman with bilateral hemotympanum secondary to spontaneous epistaxis. Initial evaluation must include an audiogram and radiological imaging (computed tomography, magnetic resonance imaging, etc.). Close follow-up of the patient is necessary for reducing the risk of long-term sequelae such as cholesterol granuloma [3].

## Case report

A 51-year-old woman was referred to the emergency department with a complaint of epistaxis associated with exercise. She had been sweeping her house when she noticed the epistaxis. Her history indicated that after epistaxis had started, she went to the sink and cleaned her nose with water. She had pressed on her nose and called an ambulance. About 30 min after the start of epistaxis, an ambulance and emergency doctor arrived. The bleeding stopped while she was in the ambulance. Her blood pressure was 125/80 mmHg. She had an unremarkable past medical history and did not have coagulation diathesis or trauma/barotrauma, nor was she undergoing anticoagulant or salicylate therapy. She complained of slight hearing loss and a feeling of fullness in both ears. The physical examination was normal except for red-blue tympanic membranes and bilateral septal excoriation. There were no other petechiae or ecchymoses on the skin or mucous membranes. Her hematologic, biochemical and coagulation tests were also normal. Temporal bone fracture was ruled out by computed tomography scan.

**Figure 1:**
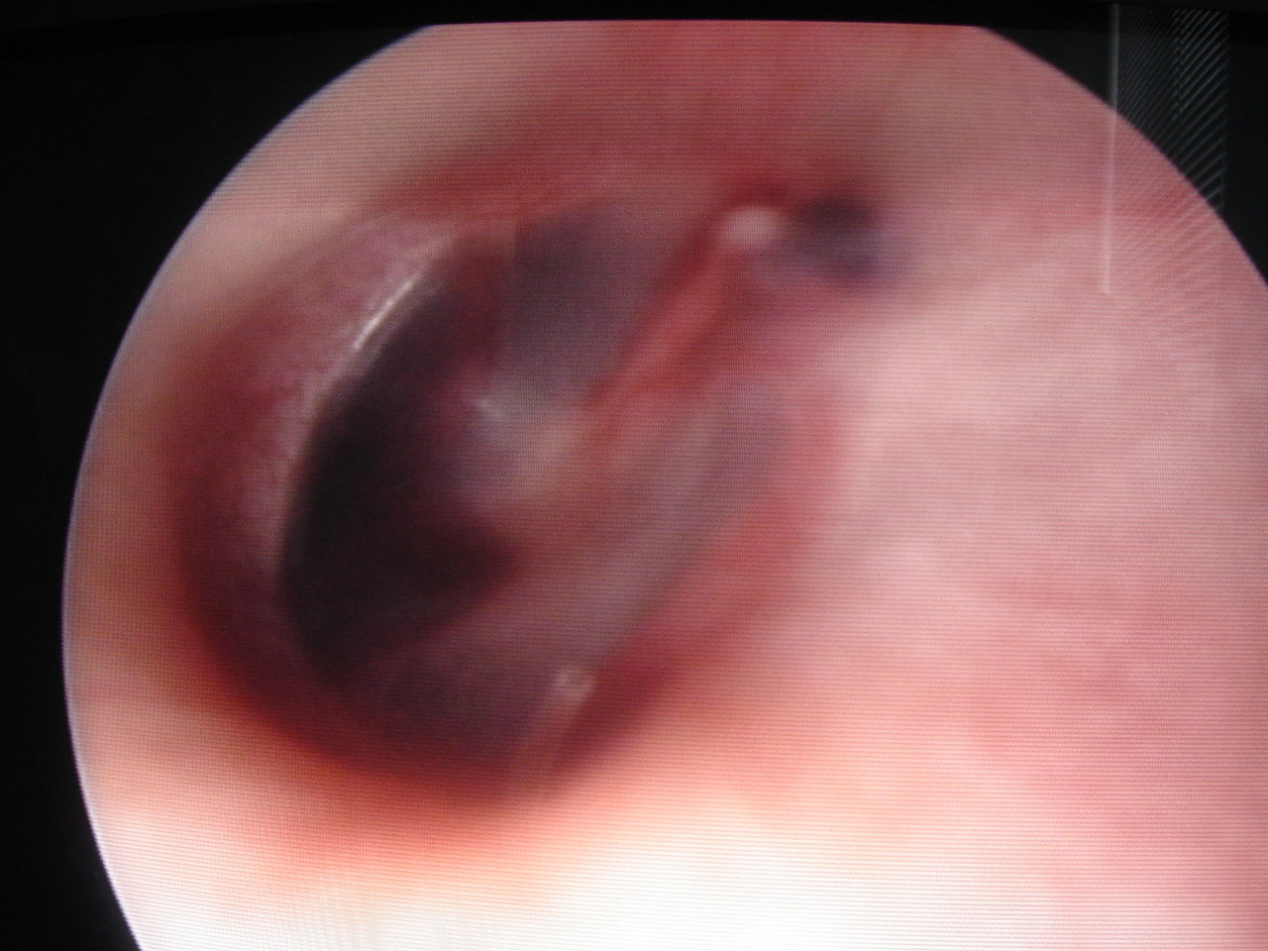
**Endoscopic view of right tympanic membrane**.

**Figure 2:**
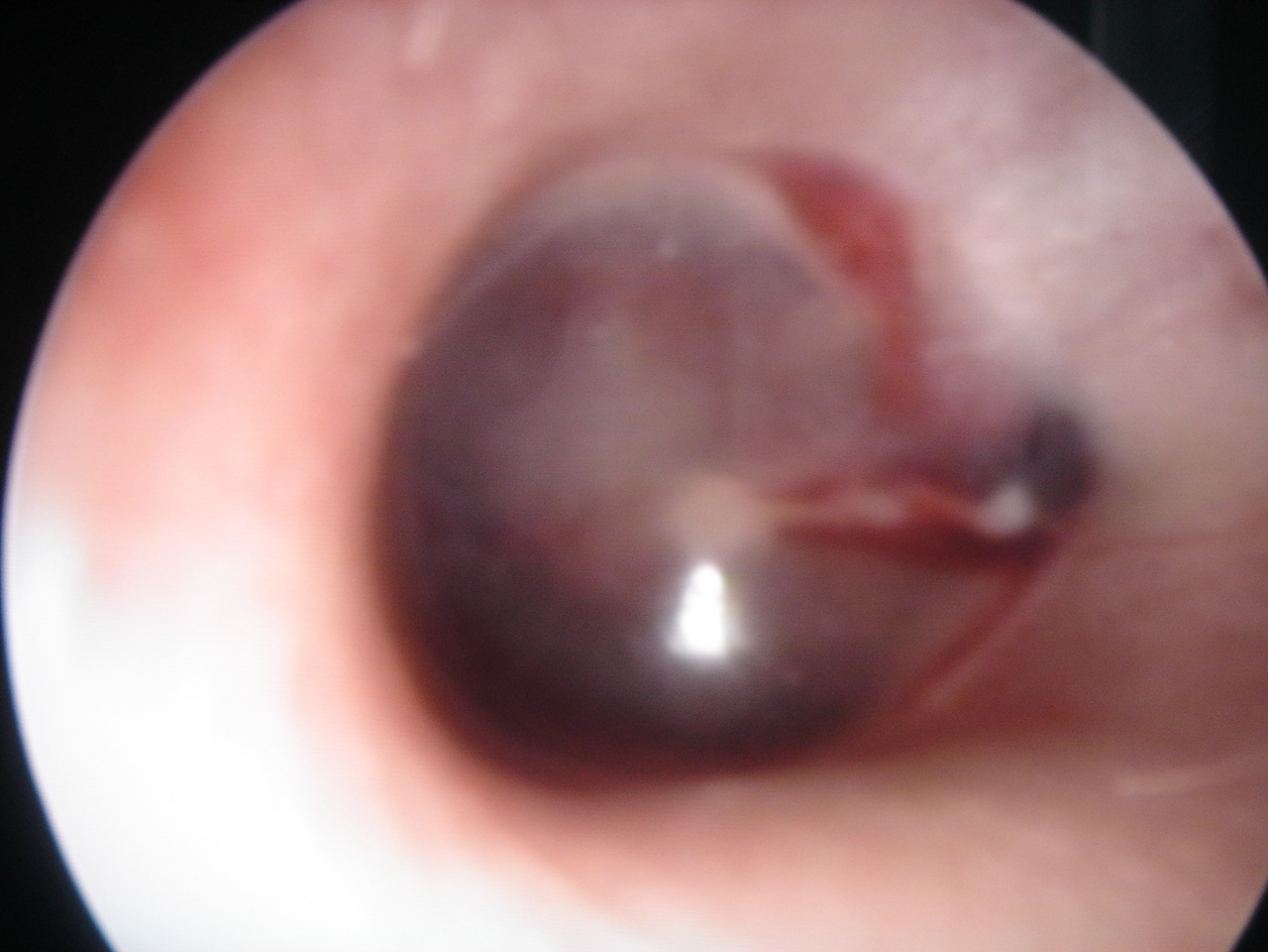
**Endoscopic view of left tympanic membrane**.

**Figure 3:**
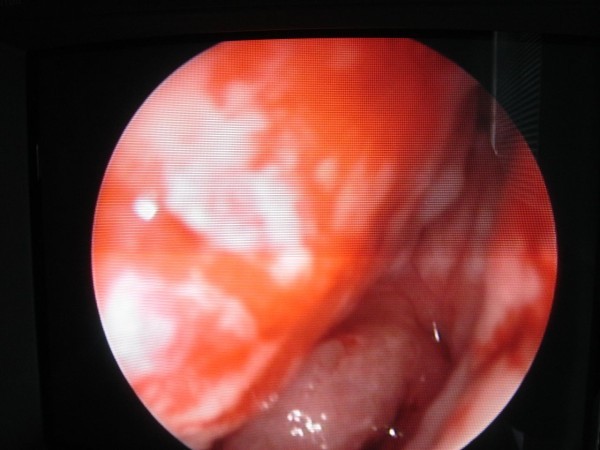
**Endoscopic view of septal excoriation**.

She was referred to the emergency department 2 days after the problem had started. In our examination, we found bilateral blue ear drums (Figures 1 and 2), inactive epistaxis and septal excoriation (Figure 3). An audiogram demonstrated moderate bilateral conductive hearing loss, and the tympanogram findings were type b (flat type). After consulting an otolaryngologist, we prescribed amoxicillin (2 g/day). Five days after starting the medication, the patient's otoscopic findings and temporal MRI were normal at the control visit.

Idiopathic or spontaneous hemotympanum is an uncommon disorder characterized by a black-blue tympanic membrane discoloration as a result of recurrent hemorrhage in the middle ear or mastoid in the presence of Eustachian tube obstruction. Initial evaluation of a blue middle ear mass includes an audiogram and computed tomography (CT) scan with intravenous contrast. CT may identify congenital vascular malformation or bone erosion due to chronic otitis media or tumors. A magnetic resonance imaging (MRI) scan is useful to distinguish hemotympanum from a vascular tumor and to avoiding angiography, which is associated with significant morbidity. Evidence suggests that secretory otitis media and spontaneous hemotympanum are different phases of the same disease process.

## Discussion

Epistaxis is common and occurs more commonly in male than female patients. Epistaxis is noted at higher incidence in older patients [4]. It is secondary to local or systemic causes. Nasal trauma (surgical, digital), foreign bodies in the nasal passage, topical sprays or dust, inflammatory nasal diseases, septal deformities, tumors and vascular aneurysms can be the local factors [5,6]. Coagulation deficits, Osler-Weber-Rendu disease and arteriosclerotic vascular diseases are possible systemic factors [5,6]. Also regular uptake of anticoagulants can cause spontaneous bilateral hemotympanum [7].

The vascular supply of nasal mucosa originates from the external and internal carotid arteries. Kiesselbach's plexus, which is on the anterior part of the septum, is the site of most epistaxis events [6]; it is also known as Little's area and is rich in vascular supply [5].

Especially temporal bone fractures, nasal packing, anticoagulant therapy, chronic otitis media and coagulation deficits are the causes of hemotympanum [8-10]. It is most often associated with temporal traumas rather than nasal packing [1], but occasionally nasal packing, which can lead to peritubal lymphatic stasis, is a cause of hemotympanum [11]. Dysfunction of the Eustachian tube is thought to be the reason for spontaneous hemotympanum secondary to epistaxis [1]. In the case presented here, there was no history of nasal packing, so retrograde blood reflux to the Eustachian tube could have been the cause because there was a history of nasal pressure that could have caused reflux to the Eustachian tubes.

Computed tomography or magnetic resonance imaging is necessary for making the differential diagnosis concerning the etiology of epistaxis [12]. In temporal traumas a fracture line could be visible on the scan, and chronic middle ear effusion can also be seen in cases of chronic otitis media. In patients with a basilar skull fracture, there can also be facial paralysis, tympanic membrane perforation or otorrhea. In patients with chronic otitis media, retraction pockets on the tympanic membrane are also visible.

All patients with hemotympanum need close follow-up. A fluid-filled middle ear cavity may result in conductive, sensorineural or mixed hearing loss [13]. Not the type of fluid in the middle ear but rather the amount of fluid affects the rate of hearing loss [14]. To prevent persistent effusion, physicians must treat the patient with antimicrobial drugs [15]. The hearing deficits normalize after the middle ear effusion has been absorbed. Persistency of fluid may lead to permanent conductive hearing loss. Myringotomy with tube placement is needed for persistent effusions [16]. All patients with hemotympanum must be followed up closely to ensure resolution.

## Conclusion

Generally temporal bone fractures, nasal packing, anticoagulant therapy, chronic otitis media and coagulation deficits are the causes of hemotympanum. However, infrequently epistaxis is the causative factor. In patients with spontaneous hemotympanum secondary to epistaxis, emergency doctors need to work with otolaryngologists for close follow-up. Physicians must remember that to prevent long-term sequelae of persistent hemotympanum, myringotomy may be required.

## Consent

Written informed consent was obtained from the patient for publication of this case report and accompanying images.

## Competing interests

The authors declare that they have no competing interests.

## Authors' contributions

VF intervened the patient in the emergency department. KO and FK were conceived of the study, and participated in its design and coordination. All authors read andapproved the final manuscript.
